# Excited State-Specific CASSCF Theory for the Torsion of Ethylene

**DOI:** 10.1021/acs.jctc.4c00212

**Published:** 2024-06-07

**Authors:** Sandra Saade, Hugh G. A. Burton

**Affiliations:** † Yusuf Hamied Department of Chemistry, University of Cambridge, Lensfield Road, Cambridge CB2 1EW, U.K.; ‡ Department of Chemistry, Physical and Theoretical Chemical Laboratory, University of Oxford, South Parks Road, Oxford OX1 3QZ, U.K.

## Abstract

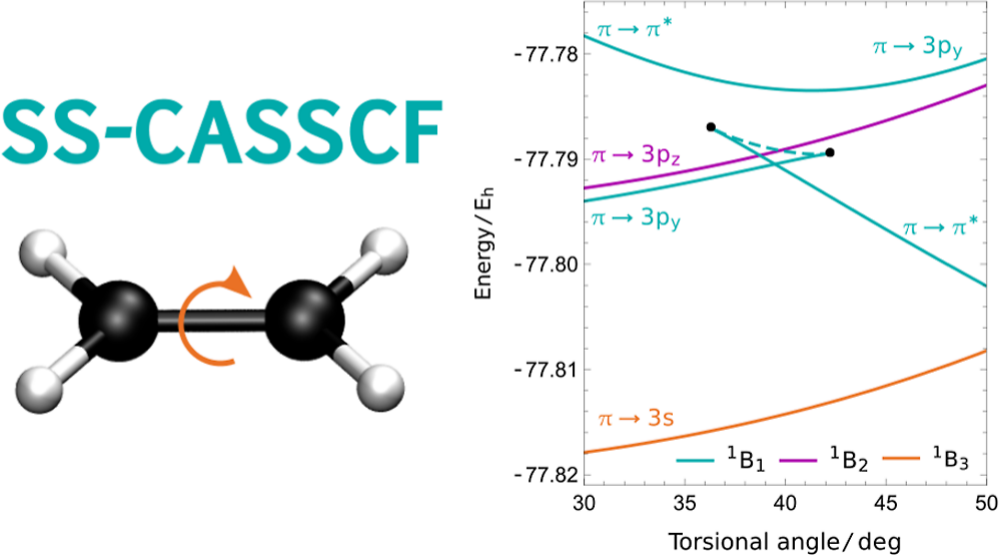

State-specific complete active space self-consistent field (SS-CASSCF) theory has emerged as a promising route to accurately predict electronically excited energy surfaces away from molecular equilibria. However, its accuracy and practicality for chemical systems of photochemical interest have yet to be fully determined. We investigate the performance of the SS-CASSCF theory for the low-lying ground and excited states in the double bond rotation of ethylene. We show that state-specific approximations with a minimal (2e,2o) active space provide comparable accuracy to state-averaged calculations with much larger active spaces, while optimizing the orbitals for each excited state significantly improves the spatial diffusivity of the wave function. However, the incorrect ordering of state-specific solutions causes excited state solutions to coalesce and disappear, creating unphysical discontinuities in the potential energy surface. Our findings highlight the theoretical challenges that must be overcome to realize practical applications of state-specific electronic structure theory for computational photochemistry.

## 1 Introduction

Simulations of dynamic photochemical processes rely on faithful descriptions of ground- and excited-state energy surfaces away from molecular equilibria, but obtaining accurate and efficient predictions of electronic excitations remains a major challenge.^1^ The prevalence of open-shell ground and excited states in photochemistry means that single-reference methods, such as equation-of-motion coupled cluster^2^ and time-dependent density functional theory (TD-DFT),^3^ are generally restricted to molecular structures around the equilibrium geometry. Therefore, computational studies rely on multiconfigurational methods, usually in the form of state-averaged (SA) complete active space self-consistent-field (CASSCF) theory.^4−6^ However, state-averaging can give discontinuous energy surfaces due to “root-flipping” when electronic states cross.^7^ Furthermore, large active spaces are required to capture all relevant states, and using a common set of orbitals does not account for bespoke orbital relaxation in charge transfer and Rydberg excitations.

Alternatively, recent research has explored the “state-specific (SS)” philosophy, where higher-energy electronic solutions are used to approximate individual excited states, which formally exist as saddle points on the exact electronic energy landscape.^8^ The simplest approximation is self-consistent field (SCF) theory, where each excited state is represented by a single Slater determinant and the optimal orbitals are computed with either Hartree–Fock theory or Kohn–Sham density functional theory.^9−20^ This approach has proved to be successful for predicting double excitations, charge transfer states,^9,12^ and core excitations.^21^ However, for open-shell states away from the ground state equilibrium geometry, one must resort to symmetry-broken SCF approximations that introduce spin or spatial symmetry contamination.^22,23^ Furthermore, state-specific SCF solutions often disappear along a potential energy surface,^8,17,23−25^ creating discontinuities that prevent dynamic simulations.

A more suitable state-specific approach for open-shell ground and excited states is multiconfigurational approximations, such as excited-state mean-field theory^26,27^ or CASSCF theory.^28−32^ Compared to state-averaging, these approaches provide bespoke orbitals for each excitation, meaning that smaller active spaces can be used.^32^ Using a minimal multiconfigurational expansion to capture the key open-shell configurations is expected to alleviate the issues of disappearing SCF solutions. Previous work has shown that unphysical solutions can still arise if the wrong active space is chosen, and solutions can undergo symmetry breaking or disappear as the molecular structure changes.^32^ However, the prevalence and significance of these irregularities for excited energy surfaces in larger molecules and basis sets of photochemical interest remain to be determined, preventing a firm evaluation of the long-term viability of SS-CASSCF theory.

In this contribution, we assess the performance of SS-CASSCF theory for the excited states in the double bond torsion of ethylene, which have been the subject of numerous theoretical and experimental studies over the past 50 years (see ref (33) for an excellent overview). The low-lying states of interest include the singlet and triplet π → 3s and π → 3p Rydberg excitations, the π → π* single excitation (V), and the  double excitation (Z). In particular, there has been significant debate about whether the π → π* state has valence or Rydberg character,^33−44^ which is compounded by the near degeneracy of the Rydberg and V single excitations and the nonvertical nature of the experimental excitation.^45−49^ SA-CASSCF theory predicts the V state to be too diffuse in character,^38,43,44^ as measured by the spatial second-order moment , which is commonly attributed to the lack of dynamic correlation.^39−41^ Furthermore, Angeli has highlighted the importance of dynamic σ-polarization and subsequent orbital contraction in the V state.^42^

At the planar *D*_2*h*_ structure, the bonding π and antibonding π* orbitals transform as b_3u_ and b_2g_, respectively, where the C–C bond coincides with the *z*-axis and the molecule lies in the *yz*-plane. The ground state and π → π* open-shell singlet excitation correspond to the 1^1^A_g_ and 1^1^B_1u_ states. Following a photoexcitation to the 1^1^B_1u_ state, the molecule is believed to rotate around the C–C bond toward the twisted *D*_2*d*_ structure, before a further pyramidalization of a –CH_2_ group leads to a conical intersection with the ground state.^50−52^ Accurate excited-state energies along this torsional mode are therefore essential, but SA-CASSCF is susceptible to root-flipping.^51^ Since each state is dominated by at most two determinants, we expect a state-specific (2e, 2o) active space to give a qualitatively correct description.

In this work, we investigate the applicability of the SS-CASSCF (2,2) approach for the ground and excited states in the torsion of ethylene. We show that multiple ground state solutions can occur, and we identify suitable stationary points for the low-lying Rydberg excitations and the V and Z excited states. We find that SS-CASSCF (2,2) can provide comparable accuracy to SA-CASSCF calculations with much larger active spaces while avoiding the issues associated with state averaging such as root-flipping. On the other hand, we show that the incorrect ordering of excitations, potentially due to missing dynamic correlation or nondiffuse basis functions, can cause solutions to disappear, giving unphysical energy surfaces. Our findings highlight the promise and pitfalls of practical excited-state applications.

## 2 Theory

### 2.1 SS CASSCF Theory

Electronic states with unpaired electrons are inherently multiconfigurational and must be modeled as a superposition of multiple Slater determinants using configuration interaction (CI). The CAS approach is the most common way to choose the subset of dominant configurations required to capture this “static” electron correlation. In CASCI, a subset of relevant active orbitals are chosen, and a CI expansion is built using every possible way of arranging the active electrons in these partially occupied orbitals. The remaining inactive and virtual orbitals are fully occupied, and empty, respectively, in each configuration.^4,53^ As a truncated CI expansion, the CASCI wave function depends strongly on the choice of orbitals in the inactive, active, and virtual spaces. Therefore, the optimal wave function is usually identified by optimizing the orbital and CI coefficients self-consistently with the CASSCF approach.^4^

On each optimization step, the CASCI wave function is defined as
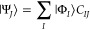
1where *C*_*IJ*_ are the CI expansion coefficients for state *J* in terms of the active Slater determinants |Φ_*I*_⟩. Variations in the CI and orbital coefficients can be represented using an exponential parametrization as
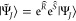
2

The anti-Hermitian operator  performs orbital rotations and is expressed in terms of the current orbitals^54−56^
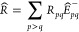
3where  is the anti-Hermitian singlet excitation operator.^57^ Similarly, the  operator transforms the CI expansion by considering the transfer operators between the target state |Ψ_*J*_⟩ and the orthogonal states |Ψ_*K*_⟩ in the CASCI space as^56^
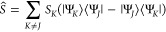
4

The energy  is then a function of the variables *S*_*K*_ and *R*_*pq*_, and the optimal CASSCF solutions are stationary points on the corresponding electronic energy landscape.

### 2.2 Computational Details

Since exact excited states are higher-index saddle points of the electronic energy landscape,^8^ we expect SS-CASSCF excited states to also be saddle points of the energy. These can be identified using second-order optimization schemes, which also accelerate convergence if there is strong coupling between the orbital and CI degrees of freedom.^56,58−60^ We employ the eigenvector-following technique^61^ to target stationary points with a particular Hessian index, as described in ref (32). For open-shell single excitations, an initial guess can be prepared by first optimizing the orbitals for a suitable configuration state function (CSF) following the framework outlined in ref (57). Once an optimal SS-CASSCF solution has been found, it can be used as an initial guess for the next molecular geometry, allowing it to be tracked across the full potential energy surface. Since the Hessian index may change along a binding curve, the mode-controlled Newton–Raphson optimizer described in ref (32) is used to reconverge solutions at each geometry without prior knowledge of the Hessian index.

All calculations are performed using an in-house computational package developed in our group, which forms an extension to PYSCF.^62^ We consider the aug-cc-pVDZ basis set,^63,64^ which includes support for diffuse Rydberg states, and the smaller 6-31G basis set.^65^ The convergence threshold is set to a root-mean-square gradient value of 10^–7^*E*_h_. SA- and SS-CASSCF(2,11) calculations were performed using the standard functionality in PYSCF.^62^ Figures are plotted using Mathematica 12.0^66^ and orbitals are visualized using the VMD software.^67^

## 3 Results and Discussion

### 3.1 Summary of SS-CASSCF (2,2) Solutions

**Figure 1:**
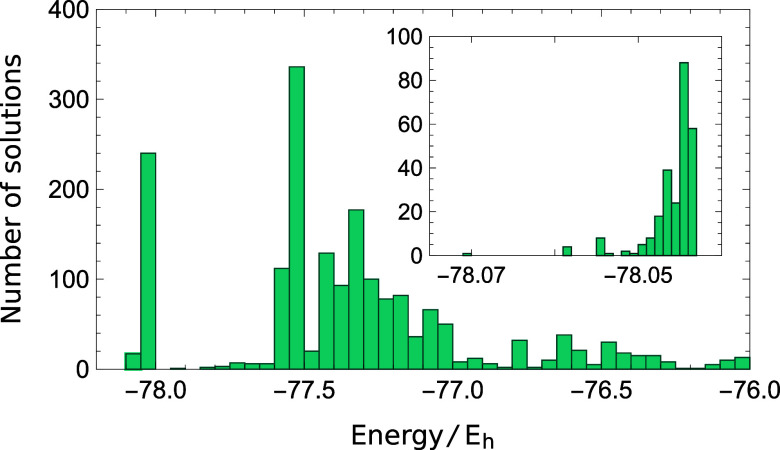
Number of SS-CASSCF (2,2) solutions identified at the *D*_2*h*_ geometry (aug-cc-pVDZ) using random starting guesses. Inset: The number of stationary points associated with the closed-shell ground state.

Using the aug-cc-pVDZ basis set, we first characterized the SS-CASSCF (2,2) solution space by starting from random MO and CI coefficients. We considered the planar *D*_2*h*_ geometry used in ref (68), which is provided in the Supporting Information. Low-energy solutions were targeted by searching for stationary points with Hessian indices between 0 and 10 using eigenvector-following. Up to 1000 random starting points were tested for each Hessian index. An extremely large number of low-energy solutions were identified, as illustrated in Figure 1, making a complete characterization of the solution space impossible.

**Figure 2:**
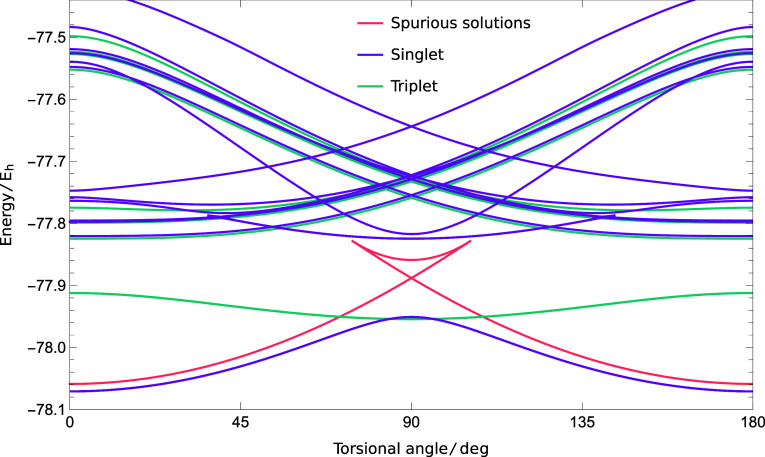
Summary of the physically meaningful singlet and triplet SS-CASSCF (2,2) solutions in ethylene (aug-cc-pVDZ) as well as the spurious local minima and index-1 saddle point (see Figure 3C).

Instead, we focused our attention on the solutions corresponding to local minima, the low-energy singlet and triplet single excitations, and the Z double excitation. Starting from a preoptimized open-shell CSF allowed suitable stationary points to be found for the (π → 3s), (π → 3p), (π → π*) excitations, among others. The  double excitation was identified by starting at the corresponding non-aufbau Slater determinant. Tracing the relevant solutions across the double bond rotation resulted in the ground- and excited-state energy surfaces shown in Figure 2.

Some solutions disappear along the torsional rotation. This disappearance can only occur if two stationary points coalesce on the CASSCF energy landscape at a pair annihilation point,^23,32^ which mathematically corresponds to a fold catastrophe.^69^ This coalescence is associated with the onset of a zero eigenvalue in the Hessian matrix of second derivatives with respect to the wave function parameters, and similar phenomena occur for multiple Hartree–Fock solutions.^18,23,32,70^ The other solution involved in the pair annihilation can be identified using a line search in the direction of the eigenvector corresponding to the zero Hessian eigenvalue, as detailed in Appendix A.

In the following sections, we characterize the local minima (Section 3.2) and the valence and Rydberg excitations (Section 3.3). Finally, we highlight how the SS-CASSCF solutions change if we use a smaller basis set that cannot describe Rydberg states (Section 3.4).

### 3.2 Multiple Local Minima

**Figure 3:**
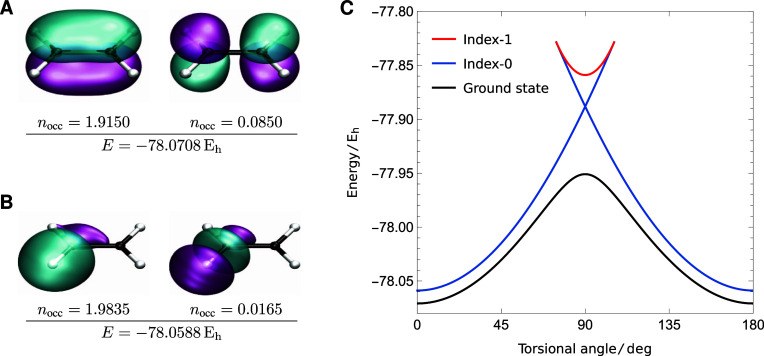
Comparison of the natural active orbitals for the SS-CASSCF (2,2) minima at the planar geometry (aug-cc-pVDZ). The global minimum (A) gives a smooth torsional barrier, while the local minima (B) give a cusp at 90° and disappear in a pair annihilation point at 106°.

Although there is only one minimum on the exact energy landscape,^8^ the SS-CASSCF (2,2) approximation yields five minima at the planar structure, corresponding to a unique global minimum and a 4-fold degenerate set of local minima. The partially occupied natural orbitals for these solutions reveal that the global minimum corresponds to the expected {π, π*} active orbitals with occupations of 1.9150 and 0.0850, respectively (Figure 3A). In contrast, the active orbitals for the local minima break the spatial symmetry and correspond to the quasi-localized C–H σ and σ* orbitals, with the 4-fold degeneracy arising from the four C–H bonds (Figure 3B). Since the true ground state is dominated by one closed-shell configuration, both active spaces include one orbital that is almost doubly occupied and one that is almost unoccupied. The active orbital with *n*_occ_ ≈ 2 can be swapped for a doubly occupied inactive orbital without significantly changing the energy, leading to multiple representations of the ground state, as described in ref (32). Therefore, in the absence of strong static correlation at the *D*_2*h*_ geometry, the different minima attempt to capture dynamic correlation in either the C–H σ or C–C π bonds.

Although both sets of minima provide a reasonable approximation to the planar geometry, choosing the right active orbitals is essential for computing physically meaningful energy surfaces.^32^ The global minimum can be followed across the full torsion to give a smooth rotational barrier (Figure 3C) because the {π, π*} active orbitals can correctly break the C–C π bond. In contrast, the energy of the C–H {σ, σ*} local minimum does not reach a maximum at 90°, and the solution eventually coalesces with an index-1 saddle point, both of which disappear in a pair annihilation point at 106°. The corresponding index-1 saddle point can be traced from 106 back to 74°, where it coalesces with a symmetry-related C–H {σ, σ*} local minimum that can be identified at the 180° planar structure. This coalescence pattern between symmetry-related local minima and a connecting index-1 saddle point is a common feature of nonlinear wave function approximations.^23,32,70^ Its presence for the local SS-CASSCF (2,2) minima in ethylene re-emphasizes the importance of selecting meaningful active spaces that can faithfully capture the static correlation across a particular chemical reaction coordinate.

### 3.3 Valence and Rydberg Excitations

The low-lying singly excited states in ethylene correspond to excitations from the π orbital to a 3s or 3p Rydberg orbital and the valence π → π* excitation. A SS-CASSCF (2,2) solution for each of the corresponding singlet and triplet excitations can be identified at the planar geometry. The orbital assignment and excitation energies are tabulated in Table 1, alongside literature benchmark values computed with extrapolated FCI^68^ (ex-FCI). In addition, we run a SS-CASSCF (2,11) calculation for the lowest-energy state of each symmetry to assess the effect of the active space size on the excited-state energy prediction. We compare against two SA-CASSCF (2,11) procedures using the same basis set and geometry. The first approach, denoted SA(5/4)-(2,11), performs a state-averaged optimization over all five tabulated singlet states or four triplet states with individual calculations for each spin sector. The second method, denoted SA(sym)-(2,11), follows the state averaging protocol outlined in ref (38), whereby individual calculations are performed for excited states of each irreducible representation, including the π → d Rydberg states, and the ground state is optimized separately. In this setup, the B_3u_, B_1g_, and B_2g_ excitations tabulated in Table 1 correspond to state-specific calculations. For reference, we also include the theoretical best estimate (TBE) from ref (33).

**Table 1:** Vertical Excitation Energies (eV) Computed with SS- and SA-CASSCF Are Compared against Literature Values and TBE^d^

state		SS-(2,2)^a^	SS-(2,11)^a^	SA(5/4)-(2,11)^a^	SA(sym)-(2,11)^a^	ex-FCI^b^	TBE^c^
1^1^A_g_		0.00	0.00	0.00	0.00	0.00	0.00
1^1^B_1u_	π → π*	8.36	8.38	8.06	8.47	7.93	8.00
1^1^B_3u_	π → 3s	6.81	6.89	6.48	6.92	7.31	7.45
1^1^B_1g_	π → 3p_*y*_	7.44	7.57	7.12	7.57	8.00	8.06
1^1^B_2g_	π → 3p_*z*_	7.49	7.58	7.14	7.58	8.00	8.11
1^3^B_1u_	π → π*	4.32	4.46	4.33	4.63	4.55	4.55
1^3^B_3u_	π → 3s	6.70	6.80	6.35	6.84	7.16	7.29
1^3^B_1g_	π → 3p_*y*_	7.40	7.54	7.08	7.54	7.93	8.02
1^3^B_2g_	π → 3p_*z*_	7.43	7.54	7.09	7.54	7.93	8.04
MSE	Rydberg	–0.51	–0.40	–0.85	–0.39	0.00	
	valence	0.10	0.18	–0.05	0.31	0.00	
MUE	Rydberg	0.51	0.40	0.85	0.39	0.00	
	valence	0.33	0.27	0.17	0.31	0.00	

a This work (aug-cc-pVDZ).

b Reference (68) (aug-cc-pVDZ).

c Reference (33) (approximately CBS).

d Errors are provided relative to the ex-FCI values with the same basis set (aug-cc-pVDZ) and geometry, taken from ref (68). Two different state-averaging protocols are considered, as described in the main text.

Compared to the ex-FCI values from ref (68), both the SS-CASSCF (2,2) and the SS-CASSCF (2,11) Rydberg excitation energies are consistently underestimated by around 0.5 eV, as shown by the mean signed error (MSE) in Table 1. The larger (2,11) active space only improves the accuracy by around 0.1 eV compared to SS-CASSCF (2,2). Since the SS-CASSCF approximation predominantly captures static electron correlation, this consistent shift suggests that there is an imbalance between the dynamic correlation in the ground and Rydberg states, supporting the findings of ref (38). In particular, the spatially compact nature of the ground state leads to regions of higher electron density and thus greater dynamic correlation than the more diffuse Rydberg states. Therefore, both SS-CASSCF (2,2) and (2,11) underestimate the ground-state energy and, by extension, the Rydberg excitation energies. In comparison, SA-CASSCF (5/4)-(2,11) provides a less accurate excitation energy than either of the SS approaches, which is expected due to the lack of specific orbital relaxation for the individual Rydberg states. The fact that the valence π → π* excitation is better approximated by using SA(5/4)-(2,11) is likely to be the result of error cancellation.

Compared to the −0.5 eV underestimate for the Rydberg excitation energies, the SS-CASSCF (2,2) approximation overestimates the V excitation energy by 0.43 eV. This overestimate can be understood because the π → π* excited state is dominated by zwitterionic resonance structures with a larger dynamic correlation energy than the ground state, which is not captured by the CASSCF approximation.^39,40,42^ This excitation energy is essentially unchanged using the much larger SS-CASSCF (2,11) active space. On the other hand, the SA(sym)-(2,11) approach provides a much greater overestimate of 0.54 eV for the valence π → π* excitation energy since the orbitals are optimized for a SA density that also includes the π → d_*xz*_ Rydberg state. Therefore, the SS protocols both provide comparable accuracy to the SA(sym)-(2,11) excitation energies, while avoiding the issues associated with SA optimization.

The quality of the wave function, and the degree of Rydberg character, can also be measured through the  value, which can vary significantly for a small change in energy.^41^ Compared to the TBEs taken from ref (33), the SS-CASSCF (2,2) approach provides estimates of  with a MUE of 2.25 *a*_0_^2^ and 1.89 *a*_0_^2^ for the Rydberg and valence states (including the ground state), respectively (Table 2). Surprisingly, the SS-CASSCF (2,11) improves the second-order moment of the valence excited states but worsens the description of the Rydberg excitations. The largest errors are obtained with the SA(5/4)-(2,11) procedure, demonstrating the advantage of using a SS approach to optimize the orbitals for each state individually.

**Table 2:** Comparison of the Second-Order Moment  (*a*_0_^2^) and Oscillator Strength *f*(au) for SS- and SA-CASSCF at the Planar *D*_2*h*_ Geometry^c^

		SS-(2,2)^a^		SS-(2,11)^a^		SA(5/4)-(2,11)^a^		SA(sym)-(2,11)^a^		TBE^b^	
state		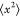	*f*	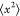	*f*	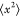	*f*	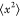	*f*	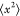	*f*
1^1^A_g_		11.68		11.74		12.00		11.74		11.78	
1^1^B_1u_	π → π*	22.52	0.298	21.29	0.356	22.45	0.364	21.66	0.355	17 ± 1	0.333
1^1^B_3u_	π → 3s	21.55	0.066	21.03	0.071	21.31	0.103	21.13	0.072	23.96	0.069
1^1^B_1g_	π → 3p_*y*_	17.89		17.64		17.59		17.64		20.38	
1^1^B_2g_	π → 3p_*z*_	18.84		18.43		18.36		18.43		21.53	
1^3^B_1u_	π → π*	11.74		11.77		12.14		11.94		11.69	
1^3^B_3u_	π → 3s	21.40		21.07		20.97		21.24		23.45	
1^3^B_1g_	π → 3p_*y*_	17.68		17.46		17.40		17.46		19.66	
1^3^B_2g_	π → 3p_*z*_	18.48		18.16		18.13		18.16		20.35	
MSE	Rydberg	–2.25		–2.59		–2.60		–2.55			
	valence	1.82		1.44		2.04		1.62			
MUE	Rydberg	2.25		2.59		2.60		2.55			
	valence	1.89		1.47		2.04		1.65			

a This work (aug-cc-pVDZ).

b Reference (33) (approximately CBS).

c Errors are provided relative to the TBE values taken from ref (33). Two different state-averaging protocols are considered, as described in the main text.

**Figure 4:**
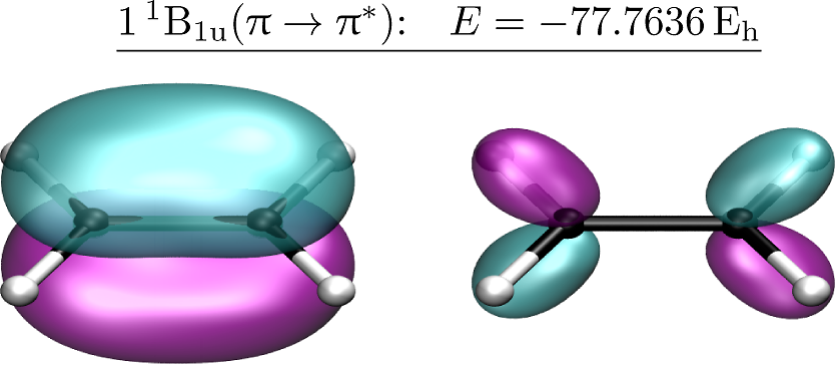
HOMO and LUMO orbitals for the 1^1^B_1u_ (π → π*) excitation in planar ethylene. The π* orbital is significantly contracted compared to Figure 3A.

Whether the 1^1^B_1u_ π → π* state has predominant valence or Rydberg character has long been disputed due to the challenge of reproducing the experimental band absorption maximum at 7.6 eV. Recent studies have confirmed that nonadiabatic effects^36,47,48^ shift this experimental value away from the vertical excitation energy that is closer to 8.0 eV,^33,49,68^ while dynamic correlation and σ-polarization are expected to cause the excited state π* orbital to contract.^42^ This spatial contraction is not easily seen in SA-CASSCF,^42−44^ and previous SA-CASSCF (2,11) calculations with an ANO basis set reported a large  value of 44.1 *a*_0_^2^.^38^ In contrast, our SS- and SA-CASSCF calculations using the aug-cc-pVDZ basis set all provide estimates between  and 22.52 *a*_0_^2^, which is much closer to the TBE (Table 2), while the SS-CASSCF (2,2) excited state visually yields a more contracted π* orbital (Figure 4) compared to the ground state solution (Figure 3A).

The relatively accurate spatial diffusivity of the SS wave functions is also reflected in the oscillator strength for π → π* excitation. Since the ground and excited states are represented with different sets of orbitals, we use the extended nonorthogonal Wick’s theorem^71,72^ implemented in the LIBGNME software package^73^ to evaluate the transition dipole moment. The SS-CASSCF (2,2) and (2,11) approaches predict the oscillator strength with deviations of −0.035 and 0.023 au from the TBE, respectively (Table 2). This accuracy suggests that the SS approach minimizes contamination from nearby Rydberg states, which have a weaker oscillator strength than the valence excitation. The π → 3s oscillator strengths are even more accurate, with deviations of −0.003 and 0.002 au for the SS-(2,2) and (2,11) approaches, respectively. By comparison, the SA(5/4)-(2,11) calculation overestimates both values by around 0.030 au.

**Figure 5:**
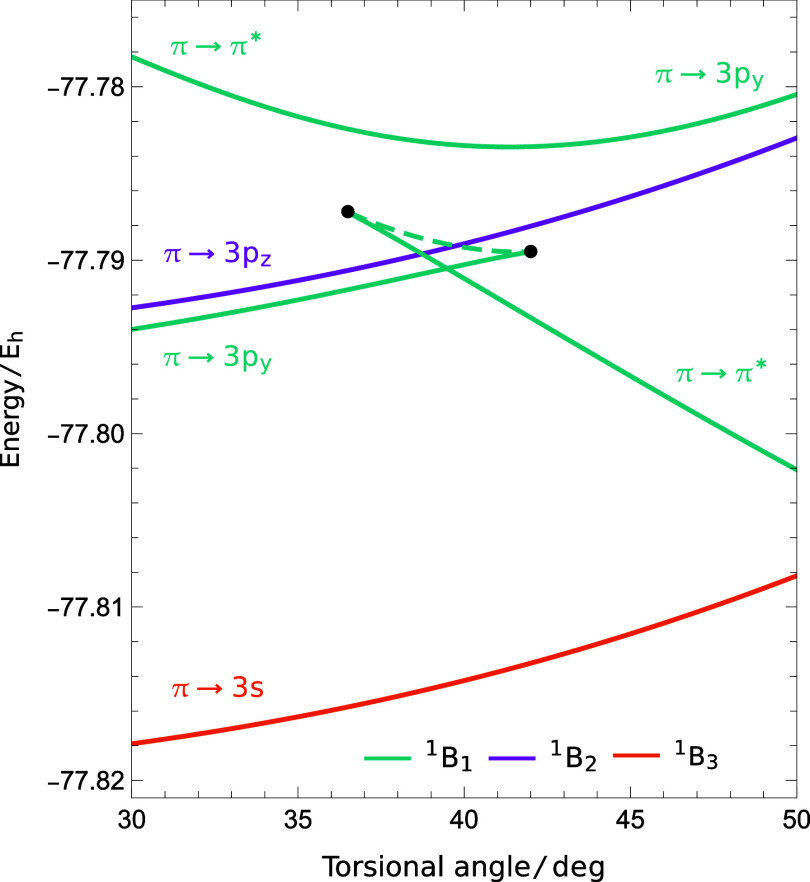
SS-CASSCF (2,2) predicts the wrong ordering for the π → π* and π → 3p_*y*_ states at the planar geometry, leading to an avoided crossing along the torsional rotation. The lower energy solution disappears at a pair annihilation point (42°) and a new discontinuous SS-CASSCF (2,2) solution emerges (37°), which represents the π → π* state at larger torsional angles. Rydberg states with different symmetries are unaffected.

Compared to the ex-FCI^68^ and TBE^33^ results, the SS-CASSCF (2,2) and (2,11) approximations both erroneously predict that the valence 1^1^B_1u_ state is higher in energy than the Rydberg 1^1^B_1g_ and 1^1^B_2g_ states at the planar geometry. This incorrect ordering has a subsequent effect on the corresponding excited-state energy surfaces along the torsional rotation. As the molecule twists away from the planar geometry, the spatial point group changes from *D*_2*h*_ to *D*_2*d*_. Under this decrease in symmetry, the planar 1^1^B_1u_ and 1^1^B_1g_ states both transform as the same ^1^B_1_ irreducible representation, meaning that they can couple through the Hamiltonian. The π → π* and π → 3p_*y*_ excited states become lower and higher in energy, respectively, eventually leading to an avoided crossing (cyan in Figure 5). We characterize this avoided crossing as unphysical since it is not consistent with the ordering of the 1^1^B_1u_ and 1^1^B_1g_ states observed in high-accuracy results.^33,68^

SS approximations are known to have unphysical solutions or coalescence points in the vicinity of avoided crossings.^17,23,32^ Here, we see that the higher energy solution, corresponding to the planar π → π* state, evolves continuously into the π → 3p_*y*_ state at the avoided crossing, as shown in Figure 5. In contrast, the lower energy solution continues to increase in energy until it eventually disappears in a pairwise coalescence point at 42°. The other solution involved in the coalescence can be followed back to 37°, where it coalesces with a third solution that corresponds to the π → π* state after the avoided crossing. These two solutions, which together form the lower part of the avoided crossing, have an unphysical state intersection around 39°. Therefore, the lower π → 3p_*y*_ solution is the only physically meaningful state that cannot be followed across the full torsional rotation, creating potential issues for the use of SS-CASSCF theory in ab initio excited-state molecular dynamics. We unsuccessfully attempted to avoid this issue using a (2e, 3o) active space that contained both the π* and 3p_*y*_. Furthermore, the same disappearance also occurs for the SS-CASSCF (2,11) solutions tabulated in Table 1, while the large number of zero Hessian eigenvalues with this active space prevents a straightforward analysis of the complementary solutions using the method described in Appendix A. On the other hand, the SS philosophy successfully avoids the more widespread discontinuities that occur in SA calculations, as seen in Figure 6 of ref (51).

Finally, we consider the double excitation , which cannot be captured by linear response formalisms such as TD-DFT. Starting from the non-aufbau Slater determinant at the planar geometry, the corresponding SS-CASSCF (2,2) solution can be identified with an excitation energy of Δ*E* = 14.46 eV and provides a continuous energy surface across the full torsional rotation (Figure 2). This state is less well covered in the literature, but benchmark values from the QUEST data set^74,75^ and ref (52) predict an excitation energy closer to 13–13.6 eV. Therefore, the SS-CASSCF (2,2) overestimates the double excitation energy, which we believe is due to the unbalanced dynamic correlation between the ground and zwitterionic excited states, as already seen for the π → π* excitation.

### 3.4 Consequences of a Nondiffuse Basis Set

The presence of low-energy Rydberg states means that diffuse basis functions are considered to be essential for accurately predicting the excited states in ethylene.^33,35,37^ We also performed SS-CASSCF (2,2) calculations using the 6-31G basis set, highlighting how the lack of diffuse basis functions can fundamentally change the pattern of SS solutions in ethylene. While the ground state exhibited a global minimum and 4-fold degenerate local minima that are directly analogous to the aug-cc-pVDZ basis, we were unable to find any physically meaningful approximations to the singly excited π → π* or the doubly excited  energy surfaces.

**Figure 6:**
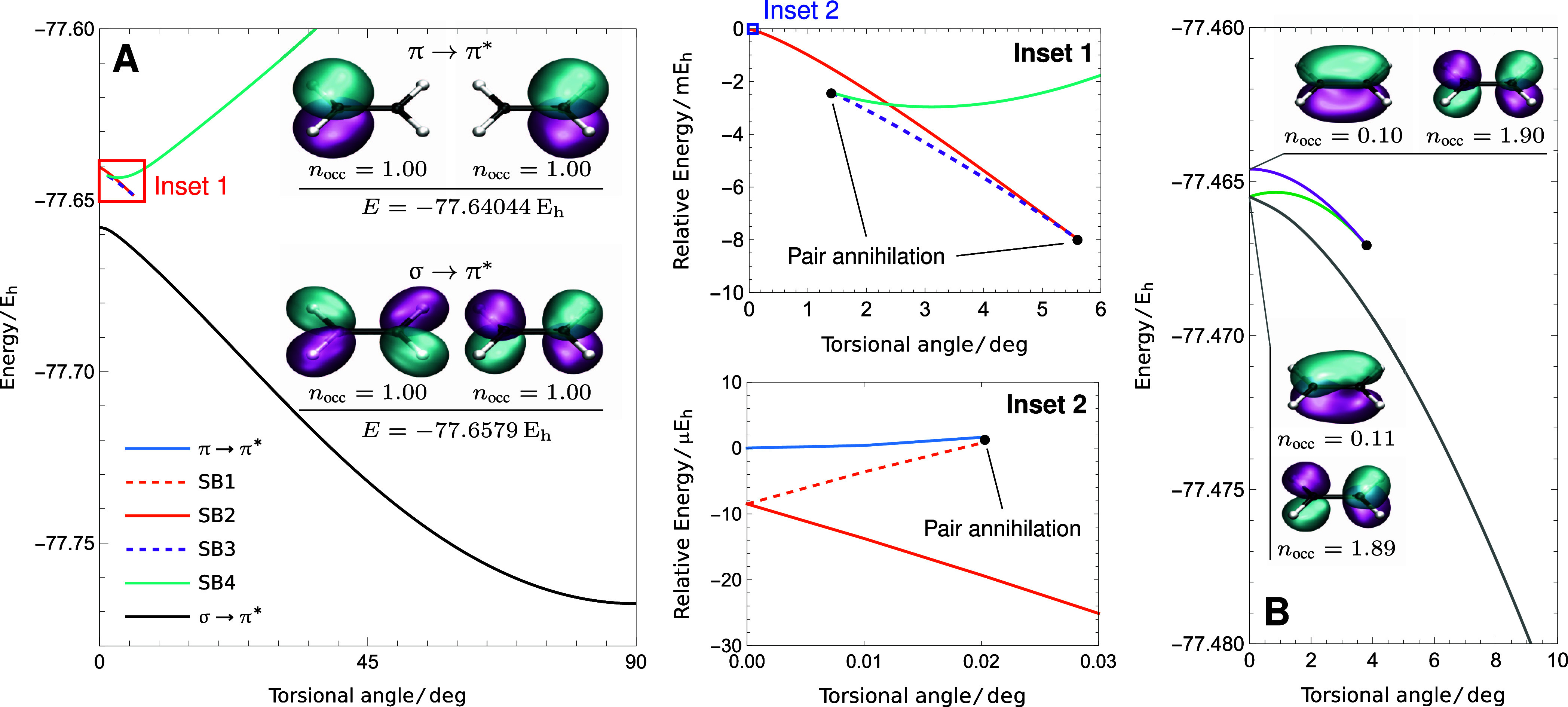
SS-CASSCF (2,2) with the 6-31G basis set does not provide physically meaningful energy surfaces for the singly excited π → π* state or the doubly excited  state. (A) This approximation predicts the wrong ordering of the π → π* and σ → π* states at the planar geometry, leading to a series of symmetry-broken (SB) solutions and an unphysical avoided crossing. (B) The symmetry-pure solution (purple) corresponding to the  excitation disappears at a torsional angle of 3.8°, giving an unphysical potential energy surface.

To target the π → π* excited state, we started the SS-CASSCF (2,2) optimization from the output of a SA-CASSCF (2,2) calculation at the planar geometry. The planar molecular structure was identified through a geometry optimization using the B3LYP functional and is provided in the Supporting Information. Starting from the SA π → π* initial guess gave a stationary point with symmetry-pure orbitals, with the natural orbitals corresponding to the localized zwitterionic configurations (Figure 6A). However, this solution only exists up to a torsional angle of 0.02°, where it disappears in a pair annihilation point (Figure 6: inset 2). A complex pattern of coalescing solutions can be found that ultimately connects the π → π* solution to another solution that emerges at 1.5°, which increases in energy for higher torsional angles (cyan in Figure 6A).

Alternatively, searching for the π → π* state at 90° yields a solution that exists all the way to 0° (black in Figure 6). However, the corresponding natural orbitals at the planar geometry indicate that this solution evolves into the ^1^B_1g_ σ → π* excitation, which is known to be higher in energy than the π → π* state using high-accuracy methods.^33^ Ultimately, the smaller 6-31G basis set results in the incorrect ordering of the ^1^B_1g_ and ^1^B_1u_ excited states because it cannot describe the diffuse character of the π → π* state, as indicated by the small  value of 12.24 *a*_0_^2^. Like the interaction between the π → π* excitation and the Rydberg states using the aug-cc-pVDZ basis, this ordering problem creates an unphysical avoided crossing that causes SS-CASSCF (2,2) solutions to coalesce and disappear as the double bond rotates, leading to catastrophic potential energy surfaces. This avoided crossing corresponds to the cyan (labeled SB4) and black curves in Figure 6A, where the cyan, orange, and dashed purple curves in Inset 1 form the upper branch with the typical structure seen elsewhere.^23,32^ The σ → π* (black) state evolves continuously into the π → π* state after the avoided crossing, while the symmetry-broken SB4 solution disappears at 1.5° in a series of pair annihilation points that eventually lead to the symmetry-pure π → π* (blue) state.

Similarly, starting from the SA states allows a symmetry-pure SS-CASSCF (2,2) solution to be identified for the  double excitation (solid purple in Figure 6B). However, this solution also disappears as the molecule is twisted and cannot be traced beyond 3.8°, where it coalesces with another solution (green in Figure 6B). This second state can be traced back to the planar geometry, where it forms a pair of degenerate solutions with natural orbitals that break the spatial symmetry (the degeneracy is lifted for nonzero torsional angles). The other degenerate solution can be followed across the full torsional mode for angles between 0 and 180° (gray in Figure 6B). However, as these degenerate solutions break the spatial symmetry and cross in energy at 0°, neither predicts a stationary point in the excited energy surface at the planar geometry. Consequently, the SS-CASSCF (2,2) approximation is not able to provide any meaningful potential energy surface for the  Z state of ethylene using the 6-31G basis, and it is vital that the basis set is sufficient for the excited states of interest.

## 4 Concluding Remarks

Excited SS-CASSCF approximations promise to overcome the challenges of SA-CASSCF theory for predicting excited energy surfaces by facilitating calculations with smaller active spaces and avoiding root-flipping discontinuities. In this work, we assessed the performance of the SS-CASSCF (2,2) approach for the valence and Rydberg excitations in the torsion of ethylene using the aug-cc-pVDZ and 6-31G basis sets. While a large number of SS-CASSCF (2,2) solutions exist, we were able to target physically meaningful stationary points for the low-lying excited states at the planar *D*_2*h*_ structure using the aug-cc-pVDZ basis set. These solutions provided excitation energies and properties for ethylene that are comparable to those of much larger SA and SS approximations. Furthermore, most of the SS-CASSCF (2,2) solutions using aug-cc-pVDZ can be continuously followed across the torsional rotation, avoiding the root-flipping problems in SA-CASSCF and the limitations of single-reference linear-response methods.

While we have only considered the excited states of ethylene, our findings support previous work^32^ showing that SS-CASSCF can be applied with only the active orbitals required for the open-shell character of each excitation. In ethylene, a (2,2) active space is sufficient to describe the torsional rotation, single excitations, and the doubly  excited state. However, larger molecules with more complex excitations or broken chemical bonds may require larger active spaces, and this must be determined on a case-by-case basis. Regardless, the findings of this work, and others,^29,32^ support the view that the SS philosophy can successfully avoid many of the issues associated with SA calculations, such as root-flipping and the large active spaces required to simultaneously predict many excitations.

The apparent imbalance between the missing dynamic correlation in Rydberg and valence excited states means that SS-CASSCF (2,2) and (2,11) fail to provide the correct state ordering in planar ethylene. This incorrect ordering of the π → 3p_*y*_ and π → π* states using the aug-cc-pVDZ basis set creates an artificial avoided crossing away from the planar geometry that manifests as a pair annihilation point, where one of the states coalesces with another unphysical solution and disappears. Therefore, there is a trade-off between coalescing solutions and root-flipping discontinuities in SS- and SA-CASSCF, respectively. Since the reference SS-CASSCF (2,2) solution mathematically disappears, these irregularities cannot be remedied by post-CASSCF correlation methods such as CASPT2,^76−78^ multireference CI,^79^ or even multistate CASPT2.^80^ Instead, we believe that a SS wave function approximation optimized in the presence of dynamic correlation will be required to stop states from disappearing. One possibility is to use a larger active space, as we tried in this work. However, larger active spaces are associated with many additional distinct CASSCF solutions if there are active orbitals with occupation numbers close to 0 or 2, making it much harder to identify a well-defined and consistent solution for each excitation. Furthermore, for the ethylene π → π* excited state, the dynamic correlation is associated with σ-polarization,^42^ which requires a full-valence active space to account for the relaxation within the σ-framework.

Finally, SS-CASSCF(2,2) calculations with the 6-31G basis set cannot capture the diffuse character of the π → π* state at all, which is predicted to be too high in energy. This error causes an artificial avoided crossing with the σ → π* excitation, and we were unable to find any meaningful energy surfaces for the π → π* or  states. These observations emphasize the importance of using sufficient basis sets for the excited states of interest and also highlight the danger of assessing SS approximations using inadequate basis sets.

Ultimately, the coalescence and disappearance of solutions remain the primary obstacle to practical excited SS calculations. These coalescence points are mainly due to the unbalanced description of different states, such as valence and Rydberg excitations. While this imbalance might be due to the lack of dynamic correlation, an alternative perspective is that the SS-CASSCF approximation simply is not the right reference for molecular excited states. Since the ethylene single excitations correspond to open-shell singlets, further restricting the wave function to a single CSF would not change our results. Instead, we believe that new wave function approximations, which explicitly include the effects of dynamic σ-polarization and orbital contraction in excited states, may provide more accurate and efficient energy surfaces for photochemistry, and we intend to pursue this direction in future work.

## Appendix A: Line Search at Pair Annihilation Points

The disappearance of a SS-CASSCF solution as the molecular structure changes indicates the existence of a pair annihilation point, which mathematically corresponds to the coalescence of two stationary points in a fold catastrophe.^69^ The Hessian index of the two solutions must differ by at most one downhill direction. For example, an index-1 saddle point can coalesce with a minimum or an index-2 saddle point. At the coalescence point itself, the two solutions become identical and one of the Hessian eigenvalues becomes zero. To find the other solution involved in this pair annihilation, we exploit the fact that the eigendirection corresponding to the zero Hessian eigenvalue points from one solution to the other if we are close enough to the coalescence point. We can then identify constrained stationary points of the energy using a line search along this eigendirection and use the one that is closest to the original solution as an initial guess for a SS-CASSCF calculation. This subsequent SS-CASSCF state will converge to the complementary solution involved in the pair annihilation. Through this procedure, we can fully map the pattern of coalescing solutions in SS-CASSCF theory.

## Supporting Information Available

Planar ethylene structure (*a*_0_) used for aug-cc-pVDZ calculations (XYZ)
Planar ethylene structure (*a*_0_) used for 6-31G calculations (XYZ)

## Supplementary Material

ct4c00212_si_001.xyz

ct4c00212_si_002.xyz
